# Novel Henipa-like Virus, Mojiang Paramyxovirus, in Rats, China, 2012

**DOI:** 10.3201/eid2006.131022

**Published:** 2014-06

**Authors:** Zhiqiang Wu, Li Yang, Fan Yang, Xianwen Ren, Jinyong Jiang, Jie Dong, Lilian Sun, Yafang Zhu, Hongning Zhou, Qi Jin

**Affiliations:** Chinese Academy of Medical Sciences and Peking Union Medical College, Beijing, China (Z. Wu, L. Yang, F. Yang, X. Ren, J. Dong, L. Sun, Y. Zhu, Q. Jin);; Institute of Pathogen Biology, Beijing (Z. Wu, L. Yang, F. Yang, X. Ren, J. Dong, L. Sun, Y. Zhu, Q. Jin);; Yunnan Institute of Parasitic Diseases, Puer, China (J. Jiang, H. Zhou)

**Keywords:** novel virus, henipa-like virus, henipavirus, paramyxovirus, Mojiang paramyxovirus, China, rats, Rattus flavipectus, viruses, zoonoses, rodents

**To the Editor:** The genus *Henipavirus* (family *Paramyxoviridae*) contains 3 established species (*Hendra virus*, *Nipah virus*, and *Cedar virus*) and 19 newly identified species, including 1full-length sequenced virus, Bat Paramyxovirus Eidhel/GH-M74a/GHA/2009 (*1*,*2*). The zoonotic pathogens Hendra virus and Nipah virus have been associated with lethal neurologic and respiratory diseases in humans, horses, and pigs (*3*–*5*). The known natural reservoirs of henipaviruses are fruit bats (*1*,*3*); these viruses have not been reported in other wild animals. We report on a novel henipa-like virus, Mojiang paramyxovirus (MojV), in rats (*Rattus* *flavipectus*) in China.

In June 2012, in Mojiang Hani Autonomous County, Yunnan Province, China, severe pneumonia without a known cause was diagnosed in 3 persons who had been working in an abandoned mine; all 3 patients died. Half a year later, we investigated the presence of novel zoonotic pathogens in natural hosts in this cave. For the investigation, we collected anal swab samples from 20 bats (*Rhinolophus ferrumequinum*), 9 rats (*R. flavipectus*), and 5 musk shrews (*Crocidura dracula*) from the mine for virome analysis.

All samples were processed by using a virus particle–protected nucleic acid purification method, followed by sequence-independent PCR amplification of extracted RNA and DNA (*6*). The amplified viral nucleic acid libraries were then sequenced by using an Illumina Genome Analyzer II (Illumina Trading, Beijing, China) for a single read of 81 bp. All raw reads were then aligned to the nonredundant protein database of the National Center for Biotechnology Information (www.ncbi.nlm.nih.gov/RefSeq/) by using BLASTx (http://blast.ncbi.nlm.nih.gov/Blast.cgi) after filtering reads as described (*6*). The taxonomy of the aligned reads was parsed by using the MEGAN4 MetaGenome Analyzer (*7*).

On the basis of the nonredundant protein alignment results, we identified 38 sequence reads that were classified as *Henipavirus* spp. However, the sequences shared low nucleotide and amino acid identities with known henipaviruses. The reads were then used for reads-based PCR to identify the partial genome of this virus. The remaining genomic sequences were determined by using genome walking. The 5′ and 3′ untranslated regions were obtained by nested PCR with combined specific primers and henipavirus-specific degenerate primers as described (*8*), and the exact sequences of the 5′ and 3′ genome termini were determined by rapid amplification of cDNA ends.

MojV shares similar features with known henipaviruses. The virus has a genome length of 18,404 nt (submitted to GenBank under accession no. KF278639), and has the characteristic henipavirus gene order: 3′-nucleocapsid (N) protein (539 aa); P/V/W/C proteins (phosphoprotein; 694 aa, 464 aa, 434 aa, 177 aa); matrix protein (340 aa); fusion protein (545 aa); attachment glycoprotein (625 aa); and large (L) protein (2,277 aa)-5′ (Technical Appendix Figure). The predicted conserved sequences between genes showed features characteristic of henipaviruses (Technical Appendix Table). The central domain of the N protein contains 3 conserved motifs common in all paramyxoviruses: QXW [I/V] X_3_K [A/C] XT, FX_2_T[I/L][R/K]Φ[G/A][L/I/V]XT, and FX_4_YPX_2_ΦSΦAMG, where Φ is an aromatic amino acid (*9*). In addition, the RNA editing site (AAAAGG) for the processing of V and W proteins conserved in the phosphoprotein gene sequences of Hendra virus and Nipah virus was found, and 6 conserved domains within the L proteins of the order *Mononegavirales* (*8*) were found in the MojV L protein.

The nucleotide identities of predicted MojV genes exhibited similarity with genes of known henipaviruses: N (53.0%–57.0% identity), phosphoprotein (37.8%–43.0% identity), matrix (59.5%–63.4% identity), fusion (47.5%–51.4% identity), attachment glycoprotein (36.6%–41.8% identity), and L (55.9%–58.6% identity) genes. Using MEGA5 (*10*), we used the phylogenetic trees based on N and L proteins to describe the evolutionary relationships between MojV and members of the family *Paramyxoviridae* (Figure). MojV clustered with the 4 members of the genus *Henipavirus* and was distant from other clusters. Thus, considering the similar genome features between MojV and other henipaviruses, we confirmed that MojV could be classified as a new species closely related to *Henipavirus* spp.

**Figure:**
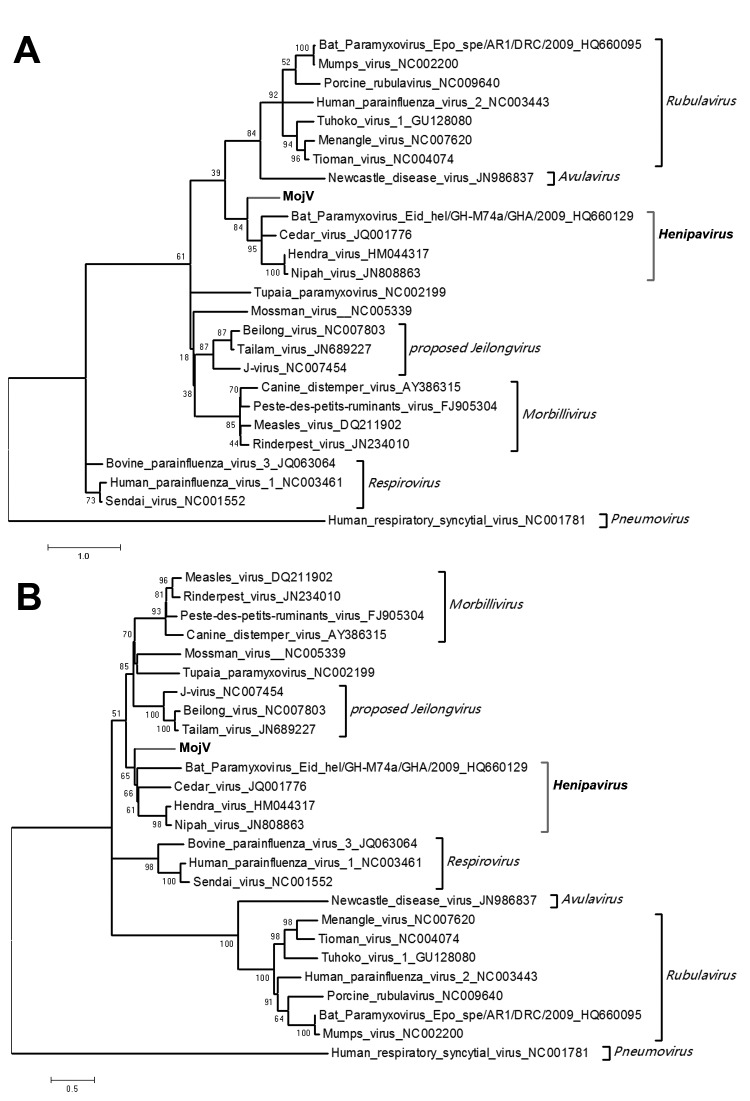
Phylogenetic trees based on the nucleocapsid proteins (A) and large proteins (B) of Mojiang paramyxovirus (MojV) and other previously reported paramyxoviruses. Bold font indicates MojV and *Henipavirus* spp. Scale bars indicate nucleotide substitutions per site.

Specific nested primer sets targeting the L gene of MojV were designed to separately re-evaluate the 34 anal swab samples and some tissue samples. Of 9 anal swab samples from the *R. flavipectus* rats, 3 were positive for MojV, and a tissue sample from 1 of the 3 MojV-positive rats was also MojV positive (tissue was not collected from the other 2 rats). All 20 samples from *R*. *ferrumequinum* bats and all 5 samples from *C*. *dracula* musk shrews were MojV negative. The 3 MojV-positive anal swab samples were cultured in Vero E6, Hep2, and BHK21 cells for virus isolation; no cytopathic effects or viral replication was detected after 2 blind subculture passages.

Our study showed the presence of a rodent-origin, henipa-like virus, MojV, in China. *R. flavipectus* rats are the natural reservoir of MojV. This finding and its context indicate that *Henipavirus* spp. viruses might infect more mammalian hosts than previously thought and that bats may not be the only hosts of henipaviruses.

Technical Appendix Genomic organization and nucleotide sequences for gene start and stop and the intergenic region of Mojiang paramyxovirus.
