# Supplementary material for: Novel Henipa-like Virus, Mojiang Paramyxovirus, in Rats, China, 2012
Source: Emerg Infect Dis. 2014 Jun;20(6):1064–6. doi: 10.3201/eid2006.131022 (PMC4036791; doi:10.3201/eid2006.131022)
Supplement: Technical Appendix — Genomic organization and nucleotide sequences for gene start and stop and the intergenic region of Mojiang paramyxovirus. [file 13-1022-Techapp-s1.pdf]

Article DOI: http://dx.doi.org/10.3201/eid2006.131022

Novel Henipa-like Virus, Mojiang Paramyxovirus, in Rats, China, 2012

Technical Appendix

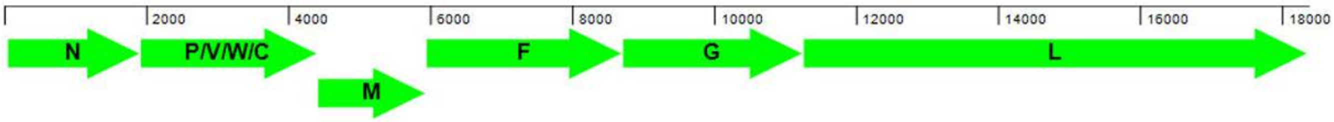

Figure. Genomic organization of Mojiang paramyxovirus. N, nucleocapsid protein; L, large protein; P/V/W/C, phosphoprotein; M, matrix protein; F, fusion protein; G, attachment glycoprotein.

Table. Conserved nucleotide sequences for the gene start, intergenic region, and gene stop of Mojiang paramyxovirus and henipaviruses*

Variable	Gene stop	Intergenic region	Gene start
MojV, gene			
/N		CTT	AGGATTCAGG
N/P	TTAAACAAAA	CTT	AGGATCCAAG
P/M	TCATAAAAAA	CTT	AGGAGTCAAG
M/F	ATATAAAAAA	CTT	AGGTGTCAGG
F/G	TTAATAAAAA	CTT	AGGAGTCAGG
G/L	TTACAAAAAA	CTT	AGGATTCACG
Consensus sequences for henipaviruses	TWAHRAAAAA	CTT	AGGANMCARG

*N, nucleocapsid; P, phosphoprotein (P/V/W/C); M, matrix; F, fusion; G, glycoprotein; L, large protein.
